# Longitudinal Hydrodynamic Characteristics in Reservoir Tributary Embayments and Effects on Algal Blooms

**DOI:** 10.1371/journal.pone.0068186

**Published:** 2013-07-11

**Authors:** Huichao Dai, Jingqiao Mao, Dingguo Jiang, Lingling Wang

**Affiliations:** 1 State Key Laboratory of Hydrology-Water Resources and Hydraulic Engineering, Hohai University, Nanjing, China; 2 College of Civil and Hydroelectric Engineering, China Three Gorges University, Yichang, China; Pacific Northwest National Laboratory, United States of America

## Abstract

Three Gorges Reservoir (TGR) is one of the largest man-made lakes in the world. Since the impoundment in 2003, however, algal blooms have been often observed in the tributary embayments. To control the algal blooms, a thorough understanding of the hydrodynamics (e.g., flow regime, velocity gradient, and velocity magnitude and direction) in the tributary embayments is particularly important. Using a calibrated three-dimensional hydrodynamic model, we carried out a hydrodynamic analysis of a typical tributary embayment (i.e., Xiangxi Bay) with emphasis on the longitudinal patterns. The results show distinct longitudinal gradients of hydrodynamics in the study area, which can be generally characterized as four zones: riverine, intermediate, lacustrine, and mainstream influenced zones. Compared with the typical longitudinal zonation for a pure reservoir, there is an additional mainstream influenced zone near the mouth due to the strong effects of TGR mainstream. The blooms are prone to occur in the intermediate and lacustrine zones; however, the hydrodynamic conditions of riverine and mainstream influence zones are not propitious for the formation of algal blooms. This finding helps to diagnose the sensitive areas for algal bloom occurrence.

## Introduction

**Figure 1:**
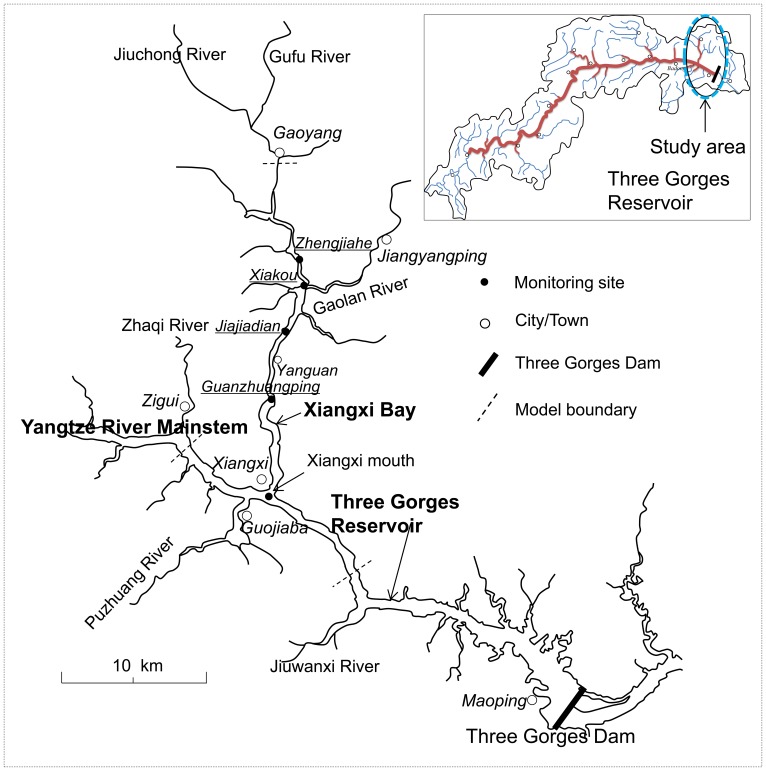
Map of Xiangxi Bay of Three Gorges Reservoir and location of model boundaries.

Three Gorges Reservoir (TGR) is a typical huge man-made lake, located in the upper Yangtze River, China. It is also one of the largest impounded reservoir systems in the world, with a normal pool level of 175 m and a total reservoir storage capacity of 39.3 billion m^3^. TGR currently faces the dual challenge of successfully performing the necessary tasks (flood control, hydropower generation, and navigation) while at the same time minimizing the negative environmental impacts [1]. Although the reservoir mainstream currently maintains the mesotrophic level, algal bloom events occur episodically in the tributary embayments [2]. For example, there were 6 bloom events observed at some embayments in 2004, 19 in 2005, and 10 from February to March 2006, respectively [3]. Among them is the representative Xiangxi Bay located not far upstream to the TGR (Fig. 1).

**Figure 2:**
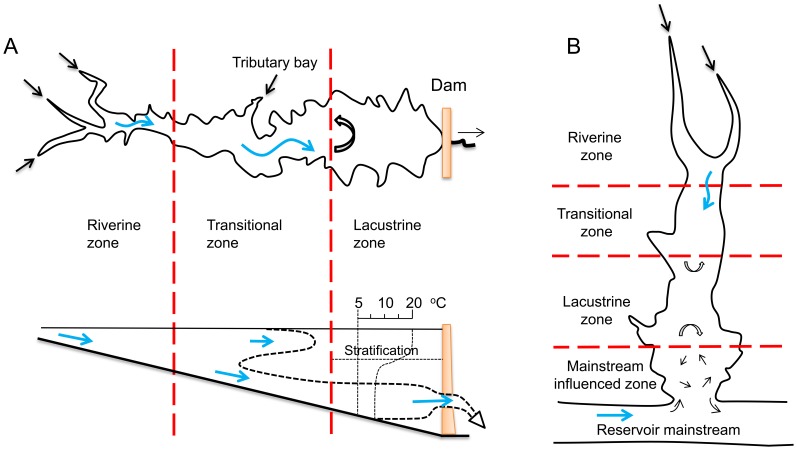
Schematic illustration of longitudimal zonation in a reservoir (the left modified from Ref [10]), and in a tributary embayment (right).

Algal bloom management must be based on a thorough understanding of the hydrodynamics, because it is commonly believed that (*i*) blooms mainly occur in eutrophic waterbodies under favorable hydrodynamic and meteorological conditions [4], [5], and (*ii*) algal bloom mitigation is determined by pollution loading and hydrodynamic conditions [6]. Spatial pattern analysis is a useful tool for reservoir ecosystem management [7]–[9]. Flow regimes of reservoirs are usually more complex than that of natural lakes or rivers, and are periodically affected by reservoir operations. Nevertheless, for a reservoir mainstream that has no large lateral tributaries, it is found that there are longitudinal gradients in the physical, chemical, and biological properties. Such a reservoir can be generally characterized as three longitudinal zones (Fig. 2a): upper riverine zone, middle transitional zone and lower lacustrine zone [10], [11]. The riverine zone is defined at the upper reach that has a narrow and channelized basin with relatively higher flow rates; the transitional zone is located at the middle reach that has a relatively broader and deeper basin with reduced flow rates; the lacustrine zone refers to the lower part immediately upstream to the dam, which has a broader, deeper and lake-like basin with lower flow rates. For example, according to the technical guideline proposed by the Ministry of Environmental Protection of China [12], the longitudinal zonation of TGR mainstream may be simply determined based on the monthly mean flow velocities: U>0.03 m/s, riverine zone; 0.03 m/s>U>0.01 m/s, intermediate zone; U<0.01 m/s, lacustrine zone.

Few studies have discussed the differences of longitudinal zonation between tributary embayments and normal reservoir mainstreams, from the point of view of hydrodynamic analysis. Over the last decade, some hydro-environment research has been carried out in the representative embayment of TGR, Xiangxi Bay. Most studies focused on the field investigation of the hydrological, hydrodynamic, and ecological conditions [13]–[15]. Some literatures studied the longitudinal patterns of phytoplankton and macroinvertebrate community [14], [16]–[17]. Whereas the general eutrophication process has been modeled [18], however, the cause-and-effect relationship between longitudinal hydrodynamic characteristics and algal bloom events are not well understood.

Numerical modeling is a powerful tool for quantitatively analyzing hydrodynamic characteristics, because it can be utilized to assess a range of alternatives (e.g., reservoir operations, discharge management, and bathymetry changes) after proper calibration and validation. It covers a wide range of models from the simple 1D Saint-Venant equations to some complex 3D turbulence models. Among the widely used and recommended hydrodynamic models are some general, flexible and dynamically-coupled frameworks [19]–[24]. Due to their flexible grid systems (e.g., curvilinear or unstructured grids), these models can be applied to different types of waterbodies in one, two, and three dimensions. In addition, for a relatively long and narrow waterbody, a 2D longitudinal-vertical model [25] is also a good choice. The models above-mentioned usually solve the vertically hydrostatic, free surface, turbulent averaged equations of water motions. Although the robustness of these models has been verified by a number of case studies, the model application still requires a good knowledge of computational fluid dynamics as well as comprehensive observational data.

The study is the first step towards developing effective algal bloom management strategies for TGR. We aim to examine the longitudinal hydrodynamic characteristics of reservoir tributary embayments, and analyze the effects on the algal blooms. Using the case study of Xiangxi Bay, a three-dimensional (3D) hydrodynamic simulation is carried out based on the general Delft3D framework. Based on the hydrodynamic analysis, a simple longitudinal zonation method is designed for reservoir tributary embayments. Through a comparative study between the hydrodynamic results and available algal bloom data, the relation between longitudinal zonation and algal bloom events is discussed.

## Materials and Methods

### Ethics Statement

No specific permits were required for the described field studies. The location studied is not privately-owned or protected in any way, and does not include a national park or other protected area of land. The field studies did not involve endangered or protected species.

### Study Area

Xiangxi River is one of the longest tributaries of TGR, located 34.5 km upstream from the dam (Fig. 1). The drainage area is around 3099 km^2^ (110°25′–111°06′E, 30°57′–31°34′N). According to the statistical data, the average annual air temperature of the study area is 16.6^o^C, and the average annual rainfall is 1016 mm [26]. It has three main subtributaries, and the average inflow discharge is 40.18 m^3^/s [26]. After the impoundment in June 2003, a river-like embayment was formed at Xiangxi River. Consequently, the flow movements in the eutrophic bay are generally weak (around 1–10 mm/s normally, and 0.1 m/s of flood peak), possibly leading to nuisance algal blooms [13], [15].

### Choice of Hydrodynamic Model

The general model framework, Delft3D, is applied and configured to simulate the flow regimes in Xiangxi Bay. We selected Delft3D as the platform because: (*i*) it is a widely accepted modeling tool for hydrodynamic simulations in rivers, lakes, reservoirs and coastal waters [27]–[28], and (*ii*) it can model the waters using curvilinear structured grids and includes the ability to simulate drying and wetting of shallow waters, which is especially suitable for river-like embayments, and (*iii*) it has been carefully validated against a series of benchmark experiments by the authors and collaborators, such as long wave propagation [29], salinity intrusion [30], stratification [31] and flushing time of waterbodies [32].

The governing equations are the unsteady 3D shallow-water equations derived from the Reynolds-averaged Navier-Stokes equations for turbulent flows, consisting of the continuity, momentum, and the hydrostatic equations. The vertical eddy diffusivity coefficient is computed following the standard two-equation *k*-*ε* turbulence model [33]. Thermal effects may play an important role in the flow regime and water quality distribution. Here, water temperature is computed by solving the transport equation which includes the net heat flux across the surface [20]. The above-mentioned governing equations are numerically solved by a combination of central and upwind spatial discretization (finite difference techniques). Specifically, the staggered Arakawa C-grid is used, where the velocity components are perpendicular to the cell faces, while the water level (pressure) is specified at the cell center. When solving the discretized equations in time, a two-step alternating direction implicit scheme is applied. More details of the numerical solution can be found in Delft Hydraulics [20].

### Data Collection

Suitable geometry, bathymetry, initial and boundary conditions are required for a river-like reservoir simulation. The geometry and bathymetry data provided by the Technology and Environmental Protection Department of China Three Gorges Corporation (CTG-TEPD) are used to supply basic information for modeling. Discharge and water level data also obtained from CTG-TEPD are used to provide the necessary initial and boundary conditions. To calibrate and validate the model, field data (i.e., water level, flow velocity, and water temperature) at the stations of Zhengjiahe, Xiakou and Xiangxi mouth (Fig. 1) during the study period are collected. The meteorological data used for water temperature computation are obtained from China Meteorological Data Sharing Service System (see http://cdc.cma.gov.cn/). In addition, to investigate the relation between hydrodynamic regimes and algal abundance of the bay, we conducted the water quality surveys in the study area, approximately on a biweekly basis at 4 sites in Spring 2005 (Fig. 1). With regard to the data quality, high frequency field observations of water levels, discharges and hydrometeorological parameters (sampling interval Δt = 1 h, 12 h, 6 h, respectively) are collected for boundary and initial conditions. However, relatively limited field data within the study area (Δt = two weeks or more) are available for calibration and validation.

### Model Configuration of Xiangxi Bay

The computational domain for Xiangxi Bay ranges from the upstream Gaoyang Town to the Xiangxi mouth which is defined at the junction between the tributary and the reservoir mainstream (∼32 km long; see Fig. 1). Considering the possible effects of the reservoir mainstream on the bay, a section of the Yangtze River is also modeled (∼18 km long; dash lines in Fig. 1). The boundary-fitted curvilinear coordinate and sigma-coordinate are adopted to fit the natural boundaries of the study area. Extensive numerical experiments were carried out using different grid resolutions, ordered from coarse to fine, to test the sensitivity of model results to horizontal model grid. The grid system eventually used includes 867×28 grid nodes for Xiangxi Bay and 359×13 grid nodes for the mainstream part. The grid cell sizes vary from 9.8 m to 58 m in the bay, and 20–70 m in the mainstream. The grid in the Xiangxi Bay has been specially refined to study the longitudinal hydrodynamic characteristics. In the vertical direction, 10 uniformly distributed sigma-layers are employed. A sensitivity test was conducted to determine the proper layers using 5, 10 and 15 sigma-layers, respectively; it is found that when modeling the thermal distribution, the cases of 10 layers can provide more detailed information and retain reasonable computational economy.

**Figure 3:**
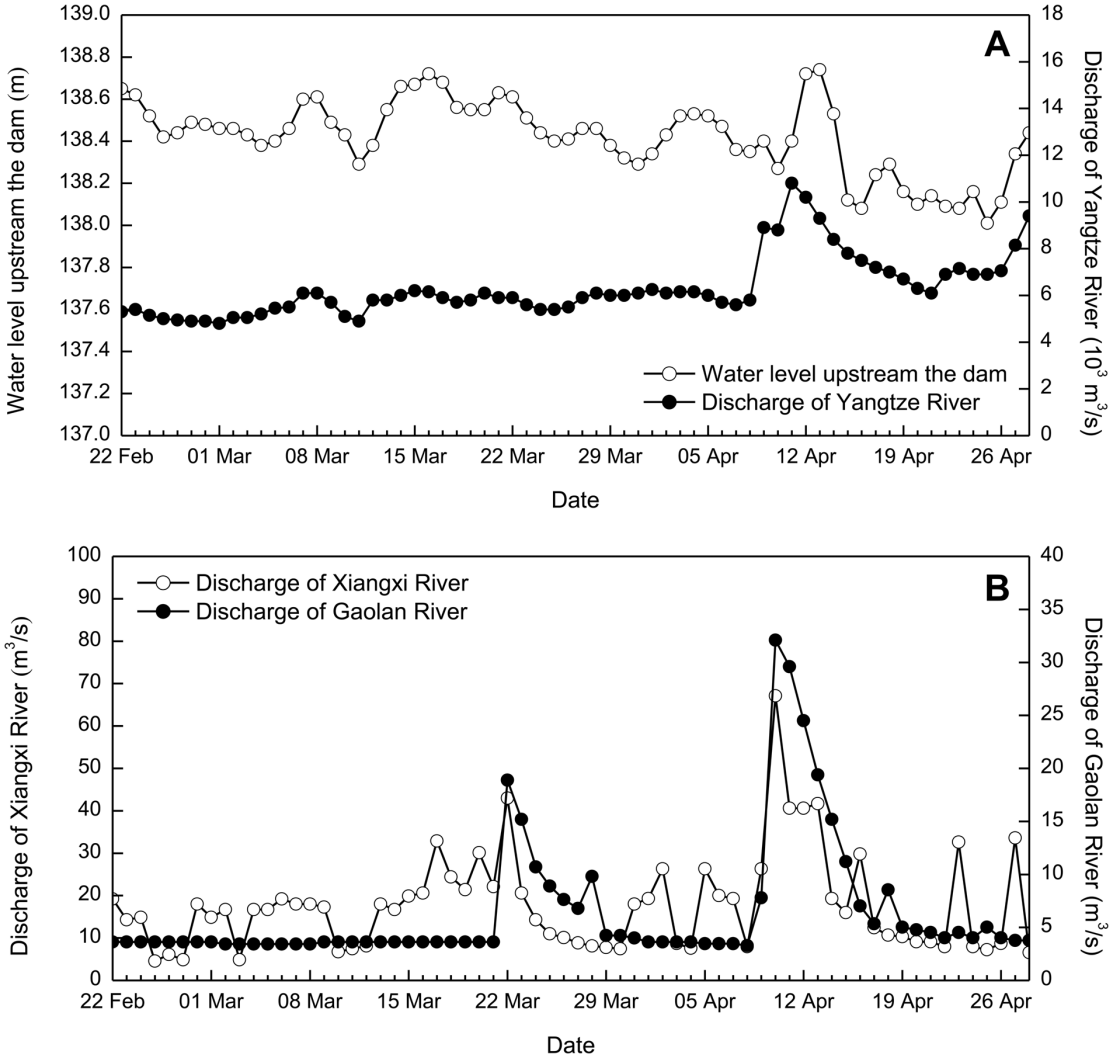
Hydrodynamic boundary conditions applied to the model: (a) observed discharges of Yangtze River and water levels immediately upstream of the dam, and (b) discharges of Xiangxi River and Gaolan River.

**Figure 4:**
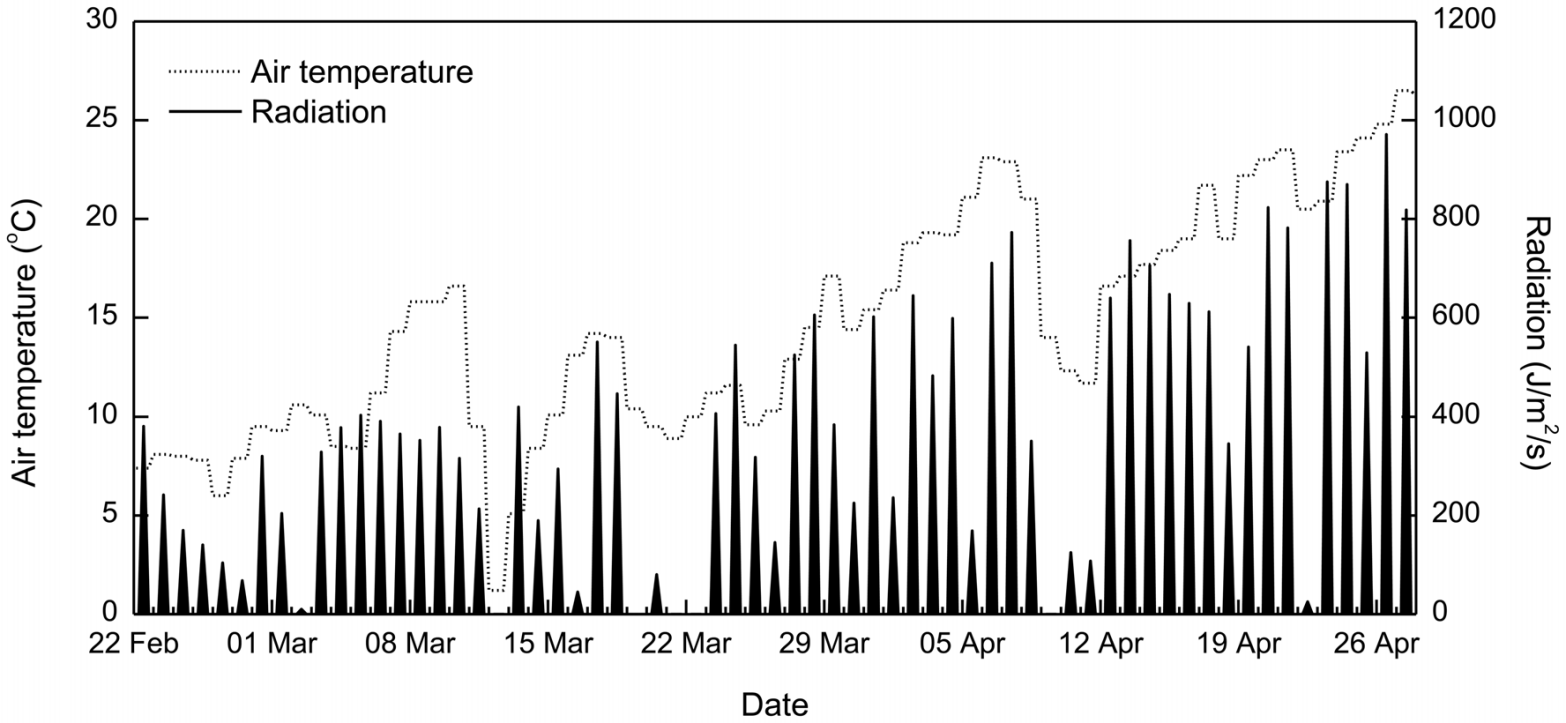
Observed air temperature and solar radiation at Xiangxi Bay during 22 Feb–28 Apr 2005.

At the upstream boundaries of Xiangxi Bay and the reservoir mainstream, the measured daily discharge data are specified, and the observed water level records immediately upstream of the Three Gorges Dam are considered to be the downstream boundary conditions (e.g., during Spring 2005; Fig. 3). A free slip condition is applied along the solid boundaries, and the transformed vertical velocity at the free surface and bottom are set to be zero. In addition, a map of spatially varying Manning roughness coefficients is calibrated: 0.03 for the mainstream, 0.026 for the bay mouth, and 0.02 for other areas. Meteorological data used for thermal computation include air temperature, radiation, pressure, humidity, precipitation and wind (speed and direction); here, the continuous air temperature and light data are provided (Fig. 4), while the mean values of other parameters are specified using the data observed at a station ∼40 km downstream from the embayment.

Considering the Three Gorges Reservoir operation is subject to the comprehensive requirements of flood control, power generation, and navigation, the water levels can vary widely during a year and may fluctuate substantially within a short time (e.g., ∼0.5 m in mid-April 2005). Therefore, during the computational process, the wetting and drying technique is used for describing the moving shores: the process of drying and flooding is represented by removing grid points from the flow domain when the water level locally falls below a certain threshold (drying cells), and by again adding grid points into the flow domain when the local water level rises above a second threshold (wetting cells). A 30-day spin-up period from “cold start” is used before the actual hydrodynamic simulation starts, for which a zero flow velocity field is assumed first while measured data are adopted at the boundaries. To reduce the spin-up time, however, the initial temperature distributions for the spin-up period are based on the spatially interpolated data, i.e., using “warm start” instead of “cold start”.

## Results

### Model Verification

To ensure the model can properly simulate the hydrodynamic characteristics of the tributary embayment, a calibration and validation procedure is performed: first, the model framework-Delft3D is fully tested against a series of benchmark experiments [29]–[32]; second, the observation of general flow pattern (in particular for the shear flow phenomenon) is then adopted to qualitatively evaluate the model performance; lastly, the model is quantitatively calibrated and verified with field data of hydrodynamics (direct validation) and water quality (indirect validation) observed in different locations and time periods.

**Figure 5:**
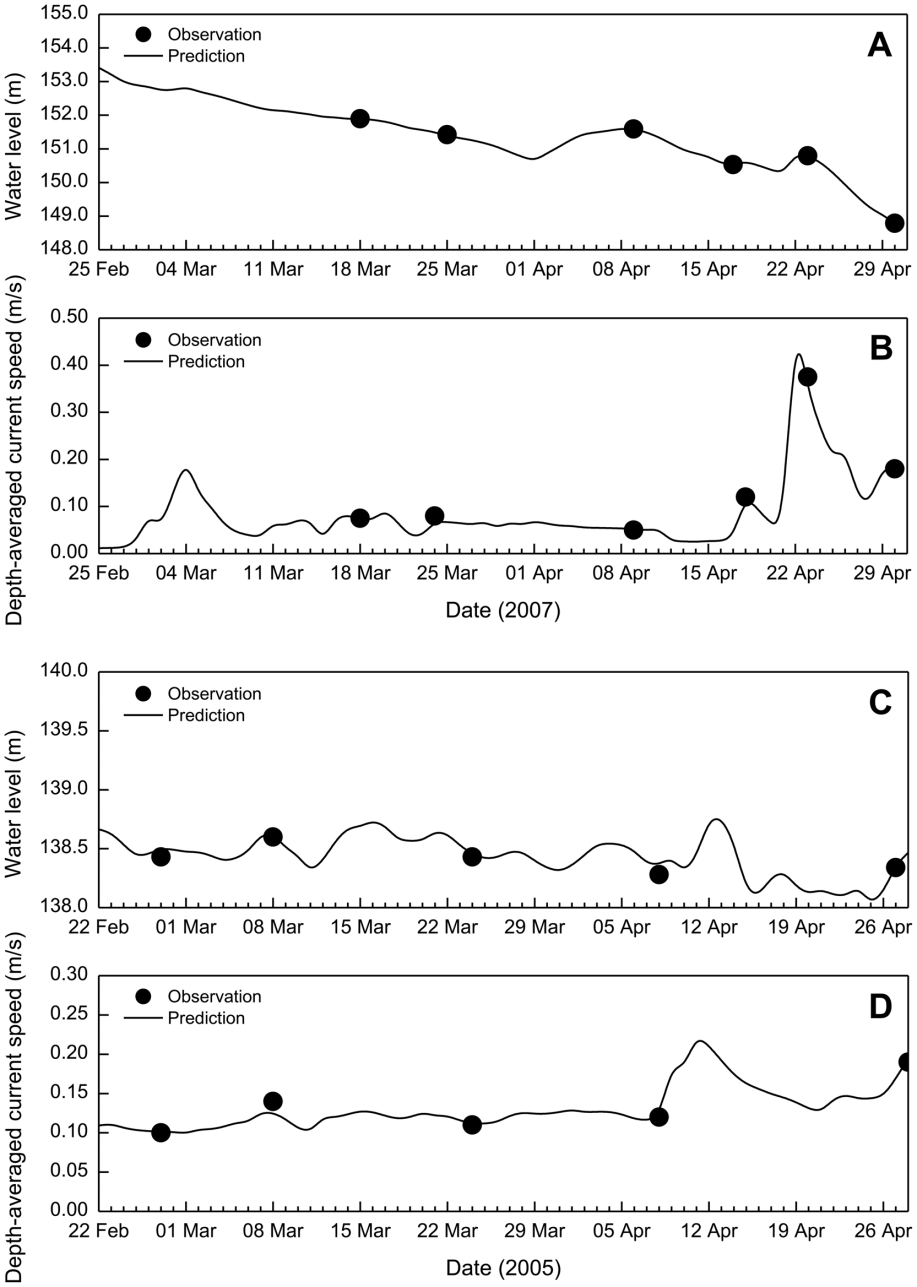
Comparison of observed and predicted water levels and depth-averaged current speeds: (a) and (b) at Station Zhengjiahe; (c) and (d) at Station Xiangxi mouth.

**Figure 6:**
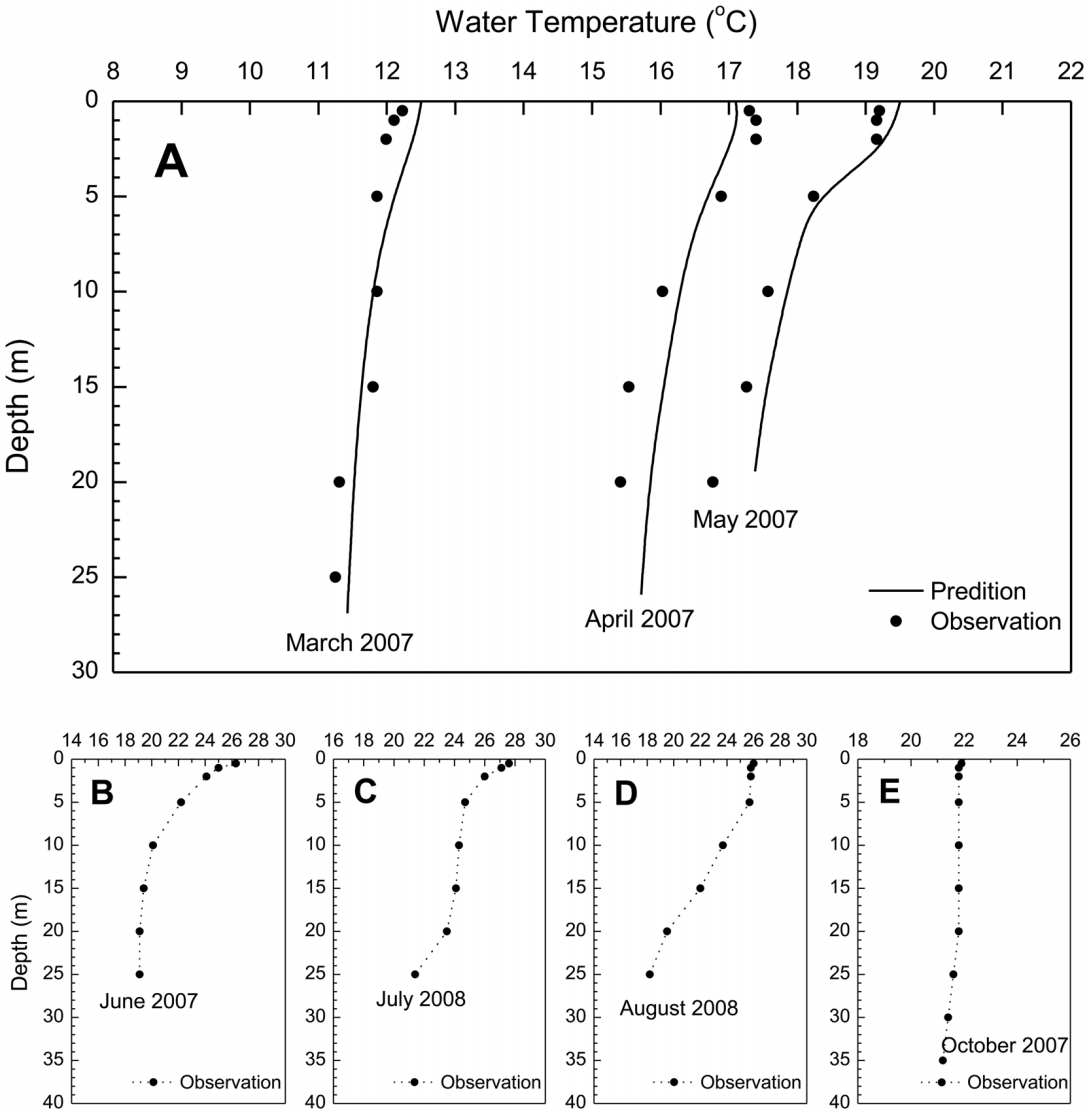
Temperature profiles at Station Xiakou: (a) comparisons between simulation and measurement in March, April, and May 2007; (b) observed values in June 2007; (c) and (d) observed values in July and August 2008 (after Ref [**34**] **); (e) observed values in October 2007.**

Two representative cases are demonstrated herein. For the step of quantitative calibration, the observed hydrodynamic process associated with the blooms during February-May 2007 is used. The observed data during February-April 2005 are then adopted to validate it again. Although the hydrodynamic conditions changes significantly from case to case, both cases show reasonable agreement of the model results and the observed data. The calibration performance is judged by comparison of the computed and measured flow velocities at a site within Xiangxi Bay (Station Zhengjiahe; Fig. 1) during Spring 2007. Fig. 5(a) and (b) show good agreement of the model with the measured water level and speed data. The mean absolute relative error (MARE = 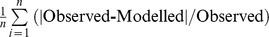) between the predicted and measured water levels is 0.024%. The MARE of depth-averaged velocity magnitude at the same site is 5.43%. Water temperature data collected at Station Xiakou (in Xiangxi Bay; Fig. 1) during Spring 2007 are used to evaluate the thermal profile results. It is shown that the model could accurately capture the onset of stratification and surface-bottom temperature differences (MARE = 1.74%; Fig. 6a). Using the calibrated coefficients while changing the boundary conditions, the computed currents in the TGR mainstream are verified with the field data measured at a station near the mouth (Station Xiangxi mouth; Fig. 1) during Spring 2005. Fig. 5(c) and (d) show good agreement of the model with the measured water level and speed data (MARE = 0.034% and 3.29%, respectively).

In summary, the model results could be accepted as a good approximation of actual hydrodynamic conditions. Note that there are some slight discrepancies for temperature simulation at some portions. As the generation of thermal stratification is caused by surface heat fluxes and weak vertical mixing, the discrepancies may be due to the inaccurate estimation of heat fluxes – e.g., caused by the quality of meteorological data or caused by the influence of turbidity on penetrative shortwave radiation. Also, it may be caused by the finite number of vertical layers in the model.

### Longitudinal Hydrodynamic Characteristics of Xiangxi Bay

With the water level reaching over 135 m, the backwater could affect the embayment up to Xiakou Town (∼25 km away from the mouth). The seasonal water temperature profiles are illustrated in Fig. 6 based on the measurements [15], [34]. It is shown that the bay remains weakly thermally stratified in the spring season (ΔT<3.0°C between surface and bottom waters), substantially stratified during the summer (ΔT>6.0^o^C), and mixed in the autumn.

**Figure 7:**
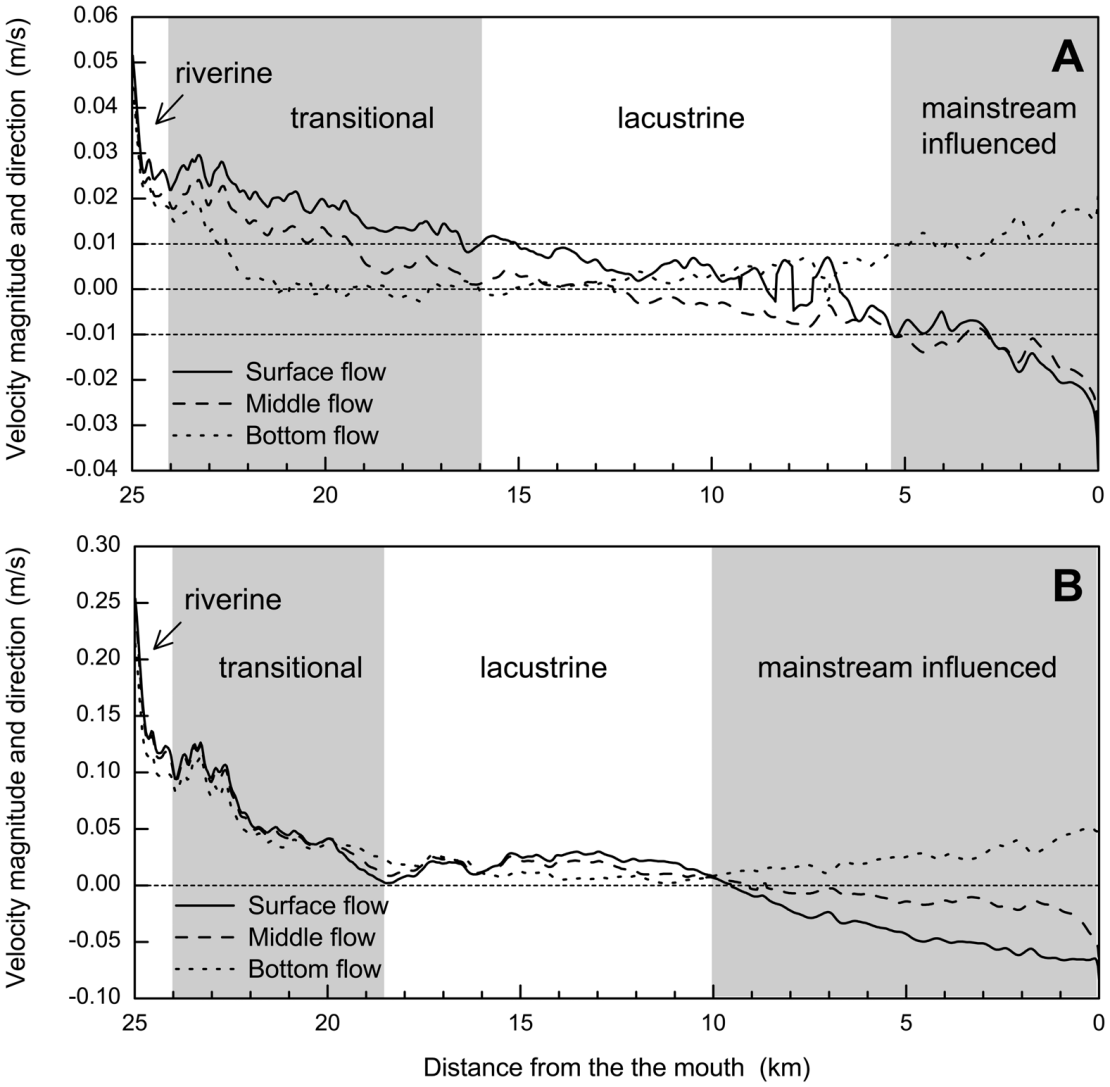
Computed surface, middle and bottom currents for Xiangxi Bay on (a) 11 March and (b) 12 April 2005, respectively.

In general, the magnitude of currents is small in the bay. Fig. 7 presents the distributions of transversely-averaged flow rates in surface, middle and bottom layers for 2 representative discharges: Case I for a relatively low inflow on 11 March 2005 (*Q* = 11.73 m^3^/s), and Case II for a relatively high inflow on 12 April 2005 (*Q* = 65.1 m^3^/s). Consequently, Case II has relatively high flow rates than Case I; the maximum velocities occur at the upstream reaches and are about 0.05 m/s for Case I and 0.25 m/s for Case II, respectively.

**Figure 8:**
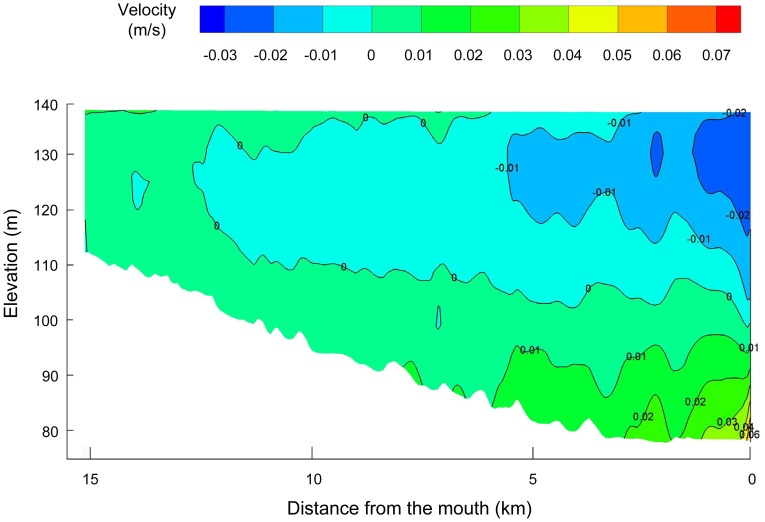
Transversely averaged velocities in the lower part of the bay: simulation on 11 March 2005 (top); observations on 15 March 2008 (middle) and 12 April 2008 (bottom).

Both cases exhibit a general decreasing trend of flow rates in the upstream portion, a quasi-stagnant flow in the middle portion, and a shear flow in the downstream portion. This flow regime is a response to the inflow discharges, the effects of reservoir mainstream and the vertical water temperature differences. In the upper reach, the circulation patterns are dominated by inflow-induced currents. However, the lower part is strongly affected by the reservoir mainstream. For example, in the lower part of the bay, the mainstream water enters the bay in the mid and upper layers through the mouth, while the embayment water flows out the mouth in the bottom layer (Fig. 8). The computed shear flow phenomena agree well with the measured vertical velocity patterns provided by Ref [34]. The slow flow formed in the middle reach stems from the interaction between the upstream and downstream effects.

The maximum influence range of the Yangtze River can extend to about 10 km upstream from the mouth (Fig. 7 and 8). This is partly because Xiangxi River has a very low flow when compared to Yangtze River; for example, during the study period from 22 February to 28 April 2005, the upstream discharges of Xiangxi River ranged from 8.17 to 99.2 m^3^/s, while the discharges of the reservoir mainstream were about 4800–10800 m^3^/s (on average 1∶300). Because the backwater can affect Xiangxi River up to around 25 km from the mouth after the impoundment of TGR, the relative contribution of the upstream inflow to the overall water movement in the tributary decreases significantly with increasing wetted cross-sectional areas. Meanwhile, the weak thermal stratification in the bay can further inhibit the vertical motion, while not restricting the horizontal movement. For example, assuming that there is a uniform temperature distribution, the model results show that only the waterbodies at the mouth (∼1 km) are directly controlled by the mainstream.

## Discussion

### Longitudinal Zonation of Hydrodynamics

As stated above, a purely river-like reservoir formed in the mainstream is often divided into three longitudinal zones (Fig. 2a). Their hydrodynamic differences lead to potentially longitudinal gradients in various components (Table 1). Such a type of zonation is caused by the dam constructed at the end of the reservoir mainstream. However, unlike a purely river-like reservoir, a tributary embayment such as Xiangxi Bay is not directly obstructed by a dam, but is strongly disturbed by the outer water (reservoir mainstream). Therefore, the longitudinal zonation of a tributary embayment would differ from the typical zonation of a purely river-like reservoir. Here, we may distinguish the longitudinal zonation of Xiangxi Bay based on the hydrodynamic modeling results. As can be seen from Fig. 7, there are nonnegligible hydrodynamic gradients along the tributary embayment: (*i*) substances in the upstream part are primarily advectively controlled (steep velocity gradient); (*ii*) the importance of advection is gradually reduced in the middle-upper reach (intermediate velocity gradient), and disappears in the middle reach (negligible velocity gradient); (*iii*) the currents close to the bay mouth tend to be stratified (Fig. 7), indicating that the hydrodynamics in the downstream part is directly controlled by the reservoir mainstream. Meanwhile, for some cases of small discharges, the longitudinal gradient variations from the middle-upper reach to the middle reach are not straightforward to quantify, because the flow speeds are fairly limited (∼0.01 m/s). As Ref [12] has propose a critical flow velocity (0.01 m/s) for lacustrine zone, it is possible to analyze the longitudinal zonation by combining longitudinal gradient variations (flow regime) with the critical flow velocity (additional criterion) if necessary.

**Table 1:** A comparison of the general properties of longitudinal zonation between normal river-like reservoirs and tributary embayments.

Properties	Normal river-like reservoirs			
	Riverine	Transitional	Lacustrine	Mainstream influenced
Location	Upstream	Midstream	Downstream	None
Width	Narrow	Intermediate	Broad	None
Depth	Shallow	Intermediate	Deep	None
Hydrodynamics	High flow	Reduced flow	Little flow	None
Turbidity	Turbid	Less turbid	Clean	None
Trophic state	More eutrophic	Intermediate	More oligotrophic	None
Productivity	Low	Intermediate	High	None
Biodiversity	High	Intermediate	Low	None
**Properties**	**Tributary embayments**			
	**Riverine**	**Transitional**	**Lacustrine**	**Mainstream influenced**
Location	Upstream	Middle-upper	Middle-lower	Mouth
Width	Narrow	Relatively broad	Relatively broad	Relatively broad
Depth	Shallow	Relatively deep	Relatively deep	Deep
Hydrodynamics	High flow	Reduced flow	Little flow	Relatively high, stratified
Turbidity	Turbid	Less turbid	Relatively clean	Relatively clean
Trophic state	More eutrophic	Intermediate	More oligotrophic	More oligotrophic
Productivity	Low	High	High	Low
Biodiversity	High	Intermediate	Low	Low

**Figure 9:**
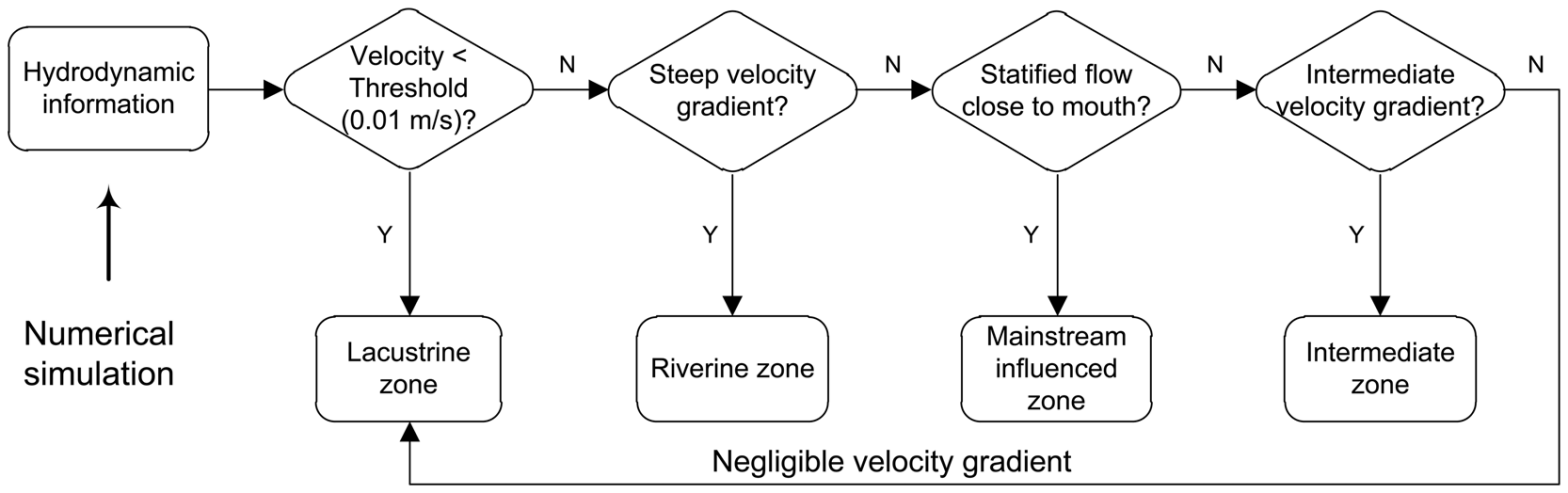
A schematic method of determining the longitudinal zones for a tributary embayment.

**Figure 10:**
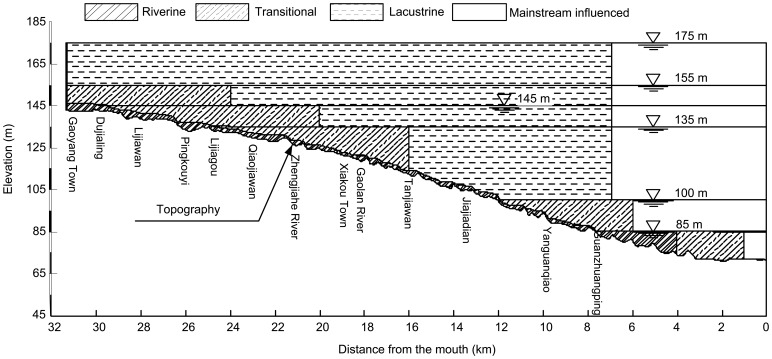
Map of longitudinal zonation under different water levels of Xiangxi Bay.

Fig. 9 illustrates the method how the longitudinal zones of a tributary embayment are determined, based on the characteristics of the surface, middle and bottom currents (e.g., Fig. 7). The critical velocity (e.g., absolute value of 0.01 m/s in a layer) may be used to distinguish the lacustrine zone if necessary, specifically for the cases of low inflows (e.g., Fig. 7a). Then, other zones are defined sequentially as follows: the upstream riverine zone is characterized with a relatively steep velocity gradient (∼10 cm/s per km); the mainstream influenced zone showing a shear flow is close to the mouth; the velocity gradients are again employed to define the transitional zone and the lacustrine zone (for the cases of high inflows, e.g., Fig. 7b). Table 1 lists the general properties of different zones between purely river-like reservoirs and tributary embayments. Their geographical similarities and differences are also graphically shown by Fig. 2. It should be noted that, longitudinal zonation can vary greatly for different inflow discharges in a given reservoir tributary embayment (Fig. 7): for the low inflow, the lacustrine and transitional zones may occupy relatively large portions of the bay; however, due to the strong influence of the mainstream for a large discharge, the extended mainstream influenced zone can compress the space of lacustrine zone and force it to move upstream. For the purposes of regional environmental management, it is more convenient to define a quasi-deterministic zonation of hydrodynamics of Xiangxi Bay. Combining the hydrodynamic model and the longitudinal zonation method above-mentioned, Fig. 10 gives a management-oriented map of longitudinal zonation on the averaged zone boundaries over all simulations using different boundary conditions. It provides a general framework to interpret the complex spatial and temporal hydrodynamics of tributary embayments, and thus may be easily used by various stakeholders. For instance, it can be observed that under different water levels after the impoundment (>135 m), the range of mainstream influenced zone is almost limited in a 7 km reach closed to the mouth. However, the lacustrine zone continues to expand as the result of increased water levels and, at the same time, the intermediate zone gradually moves upstream.

### Effects of Hydrodynamic Characteristics on Algal Bloom Risks

**Figure 11:**
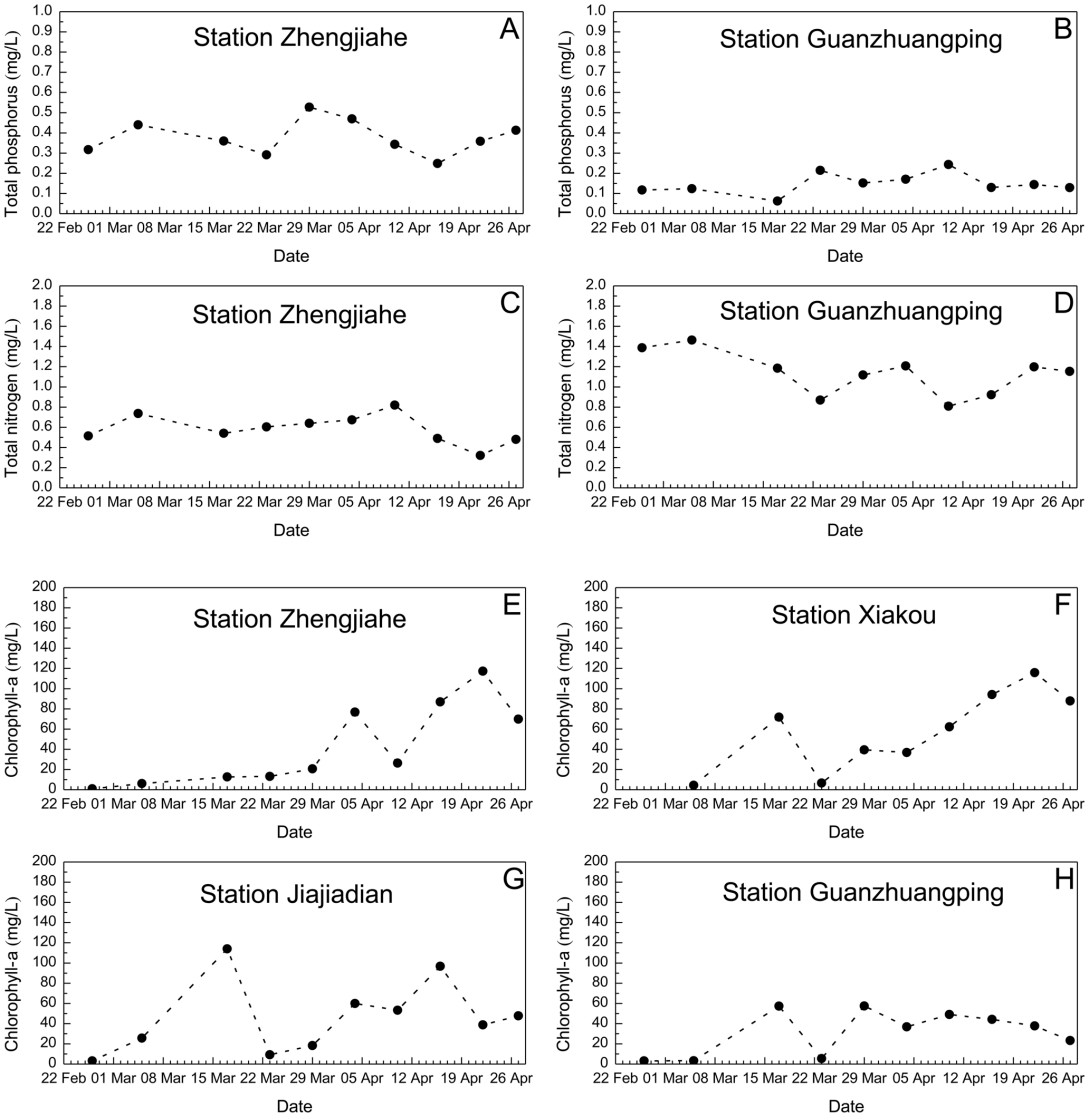
Observed water quality of TP, TN and Chla in Xiangxi Bay during Spring 2005.

Normally, an algal bloom is detected when chlorophyll-*a* (Chla) concentrations exceed a threshold (e.g., 10–12 µg/L), when cell counts are in the order of 10^3^–10^4^/mL [35]. It is interesting to estimate the algal bloom risks for different longitudinal zones, which have an early diagnostic significance in reservoir environmental management. The spatial-temporal distributions of water quality parameters that are of relevance for the algal blooms during Spring 2005 are first illustrated for several key monitoring stations (Fig. 11), of which the sites of Zhengjiahe (Z) and Xiakou (X) stand for the transitional zone, and Jiajiadian (J) and Guanzhuangping (G) represent the lacustrine zone (Fig. 1). Then, to quantify the importance of flow speeds in regulating the algal biomass, the relationship between important environmental factors and chlorophyll-*a* (Chla) is examined, using the data collected from Xiangxi Bay during Spring 2005. The method of regression analysis is used, and the differences between the relevant data sets are evaluated by unpaired t-tests.

The nutrient levels (Fig. 11a–d) indicate that the study area is in a eutrophic condition according to traditional lake trophic status indices. The observed total phosphorus (TP) concentrations are very high due to excessive phosphorus loading from the upper basin, ranging between 0.25 and 0.53 mg/L at station Z and between 0.06 and 0.24 mg/L at Station G, respectively. The mean TP value significantly exceeds the eutrophication threshold of 0.02 mg/L recommended by Ref [36]. The average total nitrogen (TN) levels are around 0.58 mg/L at station Z and around 1.13 mg/L at station G, respectively, further suggesting that the bay is suffering from excessive nutrients [37]. Therefore, algae species have a big chance to grow and bloom in the whole eutrophic area under favorable temperature, sunlight and hydrodynamic conditions.

**Figure 12:**
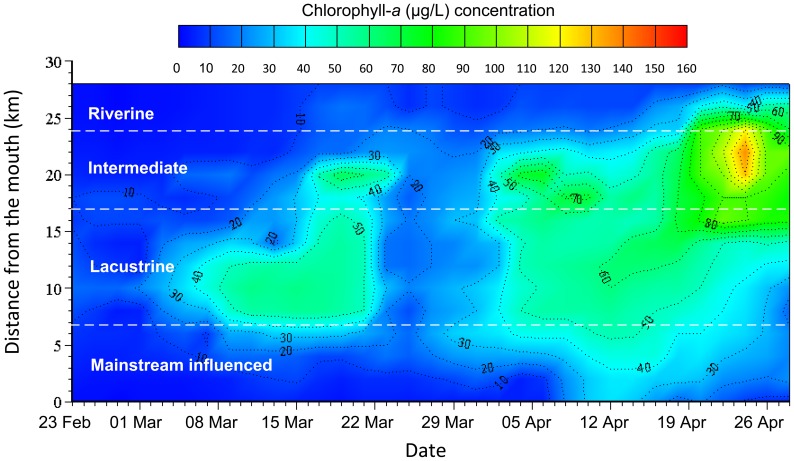
Observed spatial-temporal variations of chlorophyll concentrations for Xiangxi Bay during Spring 2005.

Across the observations at Station Z and X and J (Fig. 11e–g), there is a similar temporal pattern of the Chla concentrations: (*i*) around mid-March, the primary peak originally occurred within the domain from Xiakou to Jiajiadian; the peak concentration was about 80–120 µg/L for the middle section and decreases gradually downstream; afterwards, possibly owing to the sleet weather conditions on 12 March and during 19–22 March, this bloom disappeared quickly and did not spread to the upstream section; (*ii*) the second bloom originally occurred within the middle reach around 4 April, with the maximum value of about 80 µg/L at Station Z; (*iii*) after 2 weeks, there was another extensive bloom that occurred in the same domain, with the peak value as high as 117 µg/L; the Chla level in the lower reach was dramatically decreased (Fig. 11h), which was partially due to the relatively rapid water exchange with the reservoir mainstream outside; the bloom eventually disappeared after a week of heavy rainfall. The spatial and temporal distribution of Chla concentrations shows that, in general, there is a gradually decreasing tendency for Chla along the middle and lower bay, with the higher values occurring in the places over 15 km from the mouth (Fig. 12). In particular during the algal bloom periods, the longitudinal pattern of Chla concentrations is fairly consistent with the hydrodynamic zones determined.

**Figure 13:**
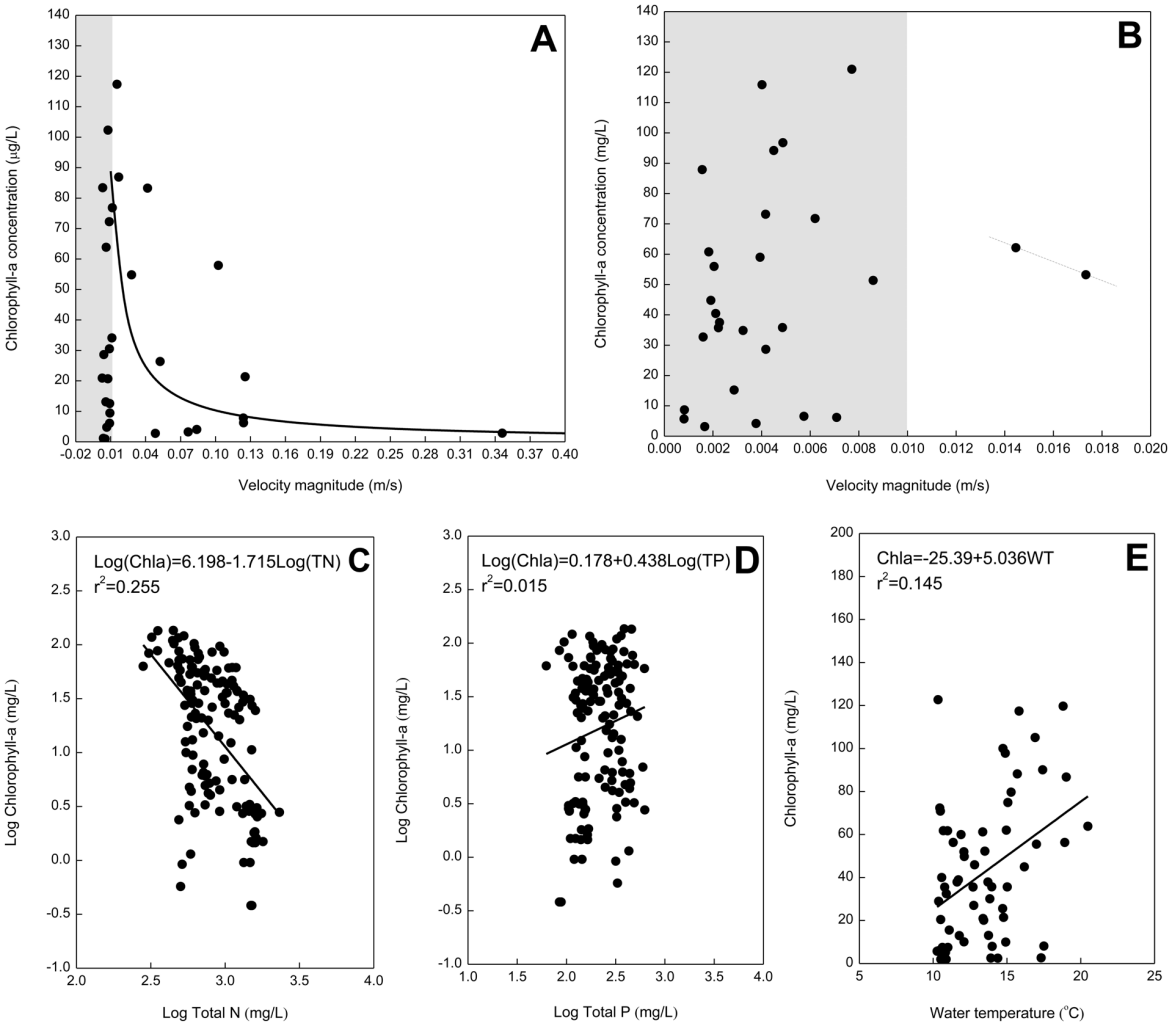
Scatter plot between the flow rates and chlorophyll concentrations for (a) the reach of 20–26 km and (b) the reach of 10–16 km from the mouth; (c) relationship between log total N vs. log chlorophyll; (d) log total P vs. log chlorophyll; (e) water temperature vs. chlorophyll.

The observed total nitrogen (TN):total phosphorus (TP) ratio varies between 0.6 and 18.9 but is generally less than 15, indicating the study area is nitrogen limited – i.e., nitrogen is often in relatively short supply compared to phosphorus. However, though there is a statistically significant relationship between log(TN) and log(Chla) (p<0.0001), log(TN) can explain only 25.5% of the variance in log(Chla) (Fig. 13c). There is no significant relationship between log(TP) and log(Chla) (p = 0.087), and the former can explain only 1.5% of the variance in the latter (Fig. 13d). Moreover, a multiple regression analysis with both TN and TP can only modestly improve the correlation with TN alone [log(Chla) = 11.98–2.7 log(TN)–1.24 log(TP), with r^2^ = 0.34]. Similarly, the relationship of water temperature (WT)-Chla is relatively significant (p = 0.001), while WT can only explain 14.5% of the variance in Chla (Fig. 13e). Analyzing the observed chlorophyll-*a* concentrations and computed flow velocities (U) during Spring 2005, it is found that almost all marked blooms (e.g., Chla >40 µg/L) are accompanied by very low flow rates less than 0.01 m/s (Fig. 13a, b), which also serves as a cross-check of the validity of the critical speed adopted for the longitudinal zonation method. Chlorophyll-*a* concentrations can sometimes still remain at low levels in the low flow environment, indicating that development of blooms may be suppressed by some external forces such as water temperature and light. When the flow speed is greater than the value of 0.01 m/s, there is a significant inverse relation between phytoplankton biomass and flow rates (Chla = 1.188U^−0.938^, with r^2^ = 0.51 and P = 0.001). Therefore, under the conditions of very high nutrient concentrations, flow velocity is considered to be the primary factor regulating chlorophyll levels.

Furthermore, we investigate all statistical locations where the algal blooms were initially observed during 2003–2009. Most blooms, 68.3%, first occurred in the intermediate or lacustrine zones; the lacustrine zone has the highest risk, 42.1% (Table 2). In particular, the reach of about 10 km long around Xiakou Town and Gaolan River is the most sensitive area. Only two bloom events were observed in the mainstream influenced zone during early June 2003 and late July 2004, respectively. Note that no algal blooms were observed in the riverine zone, indicating the very low risk of blooms for the upstream area. The analysis result suggests that algal blooms are prone to first occur in the very weakly flushed middle section though the whole embayment has been eutrophic. In other words, for a eutrophic bay, nutrient availability perhaps is not the exclusive causative factor for the algal blooms in the middle section, while the longitudinal hydrodynamic pattern plays a relatively major role in determining the high risk zones.

**Table 2:** Initial locations and biomass of algal blooms observed at Xiangxi Bay during 2003–2009.

Date	Initial location	Algal concentration (10^7^ cell/L)	Chla (µg/L)	Source
5–10 Jun 2003	Mainstream influenced zone	1.3	–	Ref [13]
Feb 2004	Whole bay	4.95	–	Ref [2]
Feb 2004	Transitional zone	4.7–5.2	–	Ref [2]
Apr 2004	Lacustrine zone	31.5	–	Ref [2]
Jun 2004	Transitional zone	5.8–9.2	–	Ref [2]
Late Jul 2004	Mainstream influenced zone	4000	–	Ref [2]
4–12 Mar 2005	Transitional zone	–	50–60	*In situ* observation
Apr 2005	Whole bay	–	60–120	*In situ* observation
Jun-Jul 2005	Transitional zone	–	60–100	Ref [13]
25 Feb 2007	Transitional zone	–	158	Ref [38]
25 Mar-9 Apr 2007	Whole bay	–	90–302	Ref [38]
6–15 May 2007	Whole bay	–	55	Ref [38]
Feb-Mar 2008	Lacustrine zone	–	25–200	CTG-TEPD
Jun-Aug 2008	Lacustrine zone	3.93–25.8	–	CTG-TEPD
21–28 Sep 2008	Lacustrine zone	–	30–60	Ref [26]
18–22 Oct 2008	Lacustrine zone	–	15–35	Ref [26]
10–24 Feb 2009	Lacustrine zone	–	1–20	*In situ* observation
13–27 Mar 2009	Lacustrine zone	–	20–55	*In situ* observation
11–28 Apr 2009	Lacustrine zone	–	20–35	*In situ* observation

## Conclusions

This study focuses on the longitudinal hydrodynamic characteristics and their effects on algal blooms in the tributary embayment of Xiangxi Bay. The spatial and temporal distributions of hydrodynamics are successfully simulated. The results show that although the currents are generally weak, there are distinct hydrodynamic gradients within the embayment. A method of determining the bounds of longitudinal zones within the tributary embayment is then developed. A tributary embayment can be generally characterized as the riverine, intermediate, lacustrine, and mainstream influenced zones. Compared to purely river-like reservoirs, there is an additional mainstream influenced zone near the mouth. The interaction between inflow discharges and the reservoir mainstream causes the existence and change of longitudinal characteristics.

The physical differences of the longitudinal zones can provide insights into algal bloom initiation in tributary embayments. The high risk zones of algal blooms are characterized by slow flow and weak gradients. The lacustrine zone represents about 42.1% and the transition zone represents about 26.3% of the observed blooms, respectively. Nevertheless, note that while there is correlation between water velocity and algal growth, there is still a multitude of factors that are believed to strongly influence algal growth, including nutrient loading, solar radiation, water temperature, water level, etc.
